# Sensitive Molybdenum Disulfide Based Field Effect Transistor Sensor for Real-time Monitoring of Hydrogen Peroxide

**DOI:** 10.1038/s41598-018-36752-y

**Published:** 2019-01-24

**Authors:** Chao Zheng, Xin Jin, Yutao Li, Junchi Mei, Yujie Sun, Mengmeng Xiao, Hong Zhang, Zhiyong Zhang, Guo-Jun Zhang

**Affiliations:** 1 School of Laboratory Medicine, Hubei University of Chinese Medicine, 1 Huangjia Lake West Road, Wuhan, 430065 P. R. China; 2 Department of Medical Laboratory, The Central Hospital of Wuhan,Tongji Medical College, Huazhong University of Science and Technology, Shengli Street Jiang’an District No.26, Wuhan, 430014 P. R. China; 3 Key Laboratory for the Physics and Chemistry of Nanodevices, Department of Electronics, Peking University, No.5 Yiheyuan Road Haidian District, Beijing, 100871 P. R. China; 4 Teaching and Research Office of Forensic Medicine, Hubei University of Chinese Medicine, 1 Huangjia Lake West Road, Wuhan, 430065 P. R. China

## Abstract

A reliable and highly sensitive hydrogen peroxide (H_2_O_2_) field effect transistor (FET) sensor is reported, which was constructed by using molybdenum disulfide (MoS_2_)/reduced graphene oxide (RGO). In this work, we prepared MoS_2_ nanosheets by a simple liquid ultrasonication exfoliation method. After the RGO-based FET device was fabricated, MoS_2_ was assembled onto the RGO surface for constructing MoS_2_/RGO FET sensor. The as-prepared FET sensor showed an ultrahigh sensitivity and fast response toward H_2_O_2_ in a real-time monitoring manner with a limit of detection down to 1 pM. In addition, the constructed sensor also exhibited a high specificity toward H_2_O_2_ in complex biological matrix. More importantly, this novel biosensor was capable of monitoring of H_2_O_2_ released from HeLa cells in real-time. So far, this is the first report of MoS_2_/RGO based FET sensor for electrical detection of signal molecules directly from cancer cells. Hence it is promising as a new platform for the clinical diagnosis of H_2_O_2_-related diseases.

## Introduction

Reactive oxygen species (ROS) play crucial roles in regulating DNA damage, signal transduction, cell proliferation and apotosis, etc.^1–4^. Hydrogen peroxide (H_2_O_2_), as a most common representative of ROS, is not only involved in several bodily disorders such as atherosclerosis, cancer, and Alzheimer’s disease, but also acts as an essential component in the physiological signaling pathways of healthy organisms, which is essential for cell growth, differentiation, migration, and immune system function^5–7^. Therefore, fast and accurate detection of H_2_O_2_ released from living cells is important for biological and clinical diagnostics application.

For detecting H_2_O_2_, there are many analytical methods, such as fluorometry^8^, spectrophotometry^9^, colorimetry^10^, electrochemical methods^11,12^, etc. Among these, the electrochemical methods for sensing H_2_O_2_ have been widely used due to their high sensitivity, fast response, and easy miniaturization. Most of electrochemical sensors involve functionalization of enzymes or proteins on the sensing interface^13–15^. Enzyme-based methods have been widely studied due to their remarkable sensitivity and specificity. However, the immobilization procedure of preparing the enzyme electrode has great influence on the biocatalytic activities of enzymes, leading to a limited stability and complicated immobilization procedure. Compared with enzymatic methods, the sensors based on nanomaterials (such as metal nanoparticles, carbon nanomaterials and metallic oxide nanostructures,) with high sensitivity and good stability brings H_2_O_2_ sensing to non-enzymatic era. Nanomaterials’ intrinsic catalytic characteristics (extremely small size and a large surface area per unit of volume) and their ability in scavenging reactive oxygen species in general can be used to mimic the catalytic activity of natural enzymes^16,17^. For example, graphene with large specific surface area, excellent electronic conductivity, and good chemical stability has been frequently reported to construct H_2_O_2_ sensing devices^18^. However, there are flaws in some ways including sensitivity, selectivity, and so on.

Recently, 2D sheet-like structure of transition metal dichalcogenides (TMDCs) has attained significant amount of interest due to their potential applications in nanoelectronics, sensing, and energy harvesting. Among these 2D TMDCs nanosheets, molybdenum disulfide (MoS_2_) is a naturally lamellar material with three atom layers (S-Mo-S) stacked together to form a sandwich structure. The unique feature renders thin MoS_2_ nanosheets considerable interest and application in catalysis, transistors, lithium ion batteries, and sensors, etc.^19–21^. Recently, MoS_2_ has been reported to directly detecting H_2_O_2_ without using enzyme and has shown intrinsic peroxidase-like activity. Lei *et al*.^22^ utilized excellent peroxidase-like activity of few layer MoS_2_ for the colorimetric detection of H_2_O_2_ with high sensitivity. Wang *et al*.^23^ fabricated a sensor for electrochemical detection of H_2_O_2_ released from cells based on MoS_2_ nanoparticles, and discovered the electrocatalytical activity of MoS_2_ nanoparticles toward the reduction of H_2_O_2_. Owing to the high sensitivity, rapid measurement, label-free detection, and compatibility with large-scale integrated circuit, field effect transistors (FETs) biosensors have attracted considerable interests^24–28^. Similarly, MoS_2_ based FET biosensor have also been applied for detecting biological molecules. Sarkar *et al*.^29^ has shown impressive sensitivity of the MoS_2_ based biosensor for detecting pH and proteins. Lee *et al*.^30^ and Jiang *et al*.^31^ utilized MoS_2_ as the sensing material for highly sensitive detection of DNA and mercury ion, respectively. Compared with zero band gap graphene, the advantage of MoS_2_ FET sensor is ascribed to the suitable band gap and high on/off ratio of MoS_2_. Although MoS_2_ can be used as the prospective candidate for the sensing channel material of FET and catalyst for the hydrogen evolution reaction (HER)^32^, so far little research is focused on utilization of MoS_2_ in the FET biosensor as a perfect catalyst toward H_2_O_2_.

**Figure 1:**
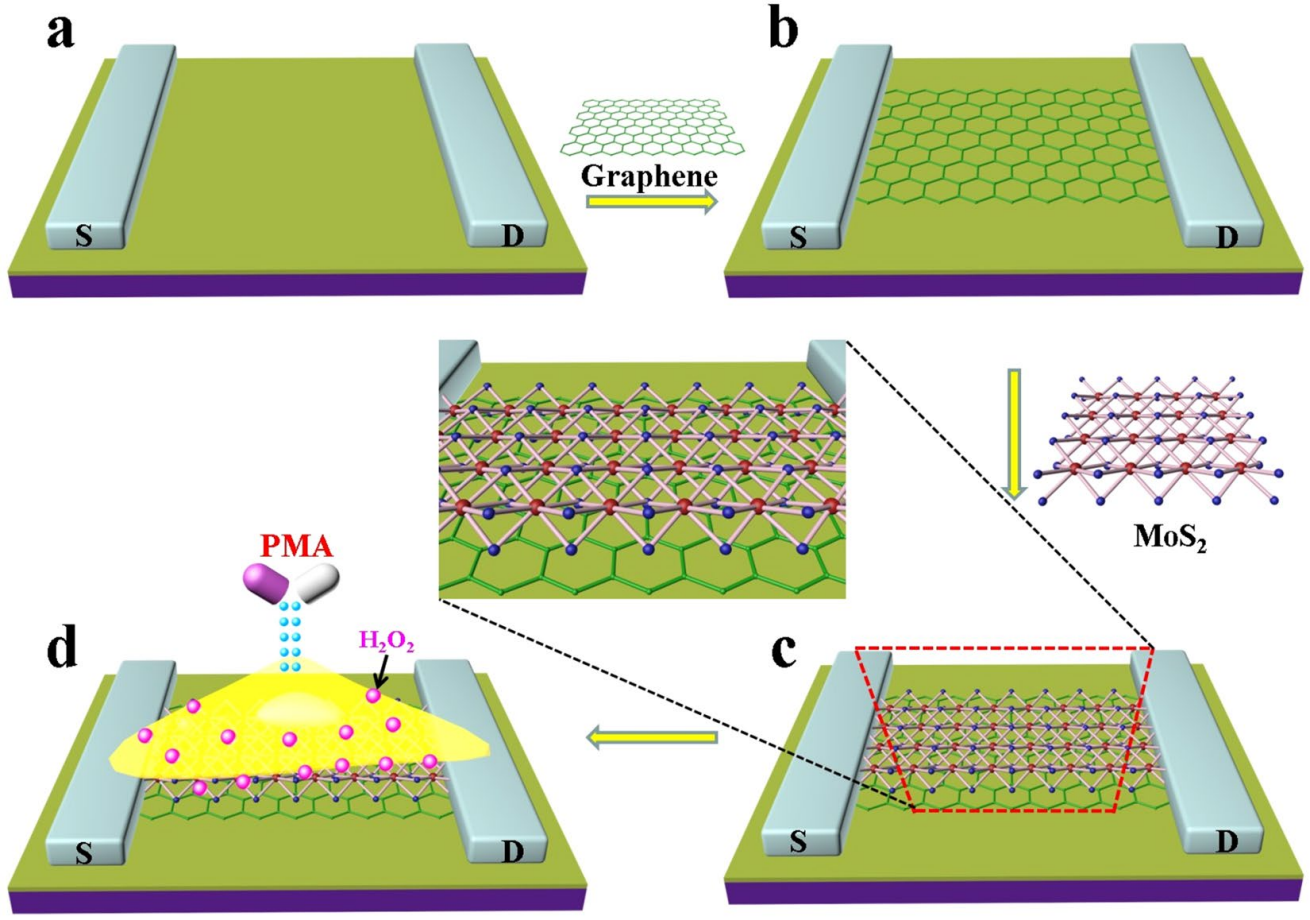
Schematic diagram of the MoS_2_/RGO FET sensor for real-time monitoring of H_2_O_2_ release from cancer cell.

In this work, we have prepared a high performance FET sensor with the introduction of MoS_2_ and reduced graphene oxide (RGO) for highly sensitive and specific detection of H_2_O_2_ from cancer cell, in which the MoS_2_ nanosheets is employed as the catalytic layer and RGO is used as the conductive layer. As illustrated in Fig. 1, a RGO sheet is drop-casted on the FET sensor surface between source and drain channel as a highly conductive bridge to facilitate rapid transport of electrons. Then the well-exfoliated MoS_2_ nanosheets are assembled on the surface of RGO. The MoS_2_ nanosheets act as an excellent catalyst and show highly catalytic activity toward H_2_O_2_. The as-prepared FET sensor responds fast and is extremely sensitive to H_2_O_2_ with the detection limit down to 1 pM. Furthermore, the MoS_2_/RGO FET biosensor is applied to monitor trace amount of H_2_O_2_ released from cancer cells.

## Results and Discussion

### Characterization of MoS_2_ nanosheets and MoS_2_/RGO FET device

**Figure 2:**
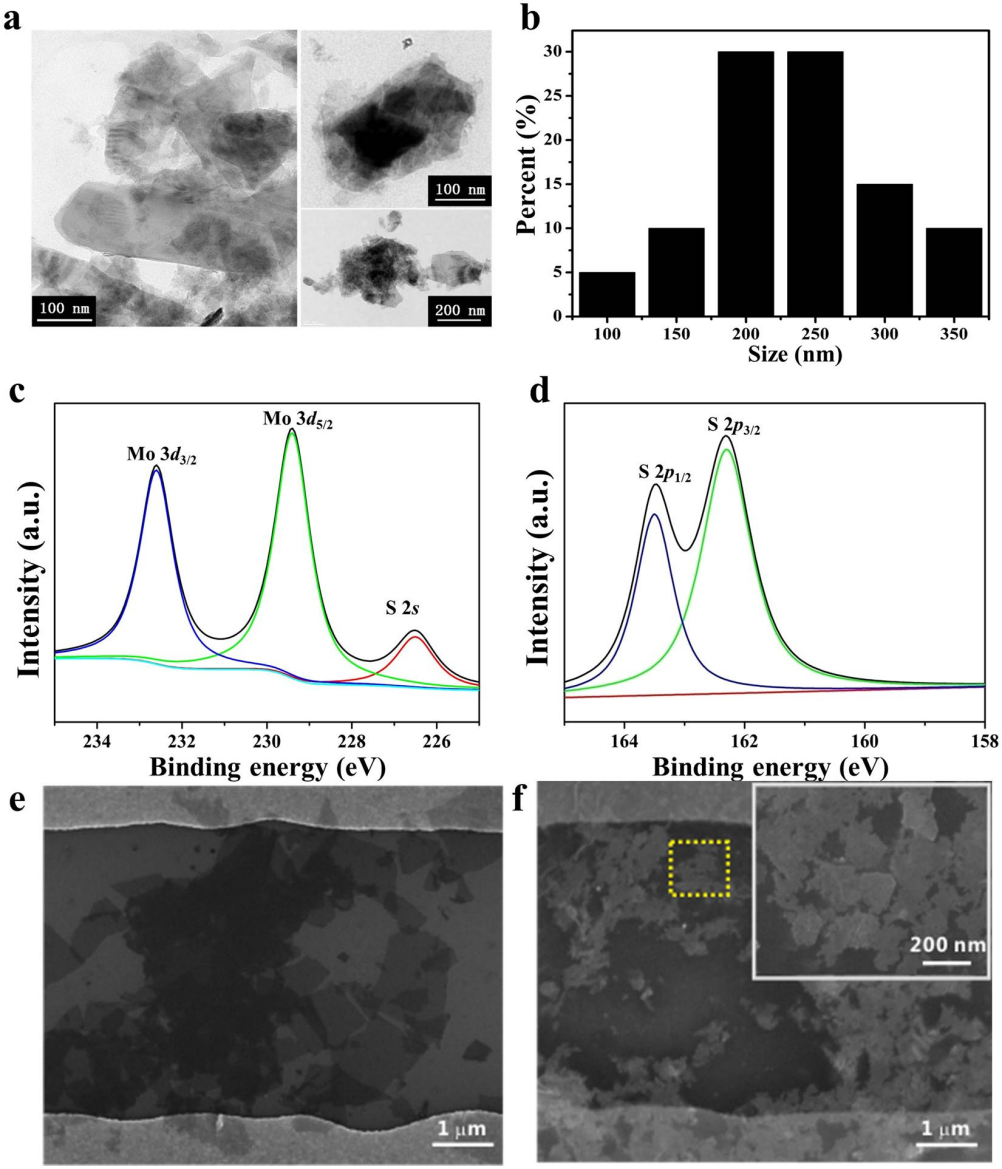
(**a**) Typical TEM images of the exfoliated MoS_2_ nanosheets. (**b**) Histogram of measured MoS_2_ nanosheet size. (**c**,**d**) XPS characterization of the MoS_2_ nanosheets. (**e**) SEM images of RGO sheet spanning across Au electrodes (**f**) SEM images of MoS_2_ nanosheets on the surface of RGO in the sensing channel.

Ultrasonication has been proved to be a simple but an effective way to exfoliate graphite, bulk MoS_2_, and some other layered materials, because ultrasonic waves generate cavitation bubbles capable of breaking up the MoS_2_ crystalline and producing MoS_2_ nanosheets^33^. As known, N-methyl-2-pyrrolidone (NMP) is an excellent solvent for exfoliating 2D layered materials^34^. So we explored NMP as the solvent to prepare MoS_2_ nanosheets by utilization of ultrasonication in the experiment. The structure and morphology of the as-exfoliated MoS_2_ nanosheets were characterized by TEM. The TEM images in Fig. 2a clearly show that the well-exfoliated nanosheets were very thin, and a histogram of measured nanosheets size in Fig. 2b indicates that the average lateral sizes of MoS_2_ were 200–250 nm.

Then, XRD was employed to characterize the MoS_2_ nanosheets. As shown in Fig. S1, the XRD spectra of bulk MoS_2_ crystals matched with the previously reported literature^35^. The typical diffraction peaks centered at 14.3° is attributed to the (002), which belongs to the bulk MoS_2_. After exfoliated, the characteristic peak disappeared, indicating the existence of lamellar form in the exfoliated MoS_2_. The results demonstrate the successful fabrication of MoS_2_ nanosheets.

As known, the MoS_2_ nanosheets contain stable hexagonal semiconducting phase (2H phase) and metastable metallic phase(1T). XPS was employed (Fig. 2c,d) to survey the spectrum of Mo and S and the surface chemical properties of the as-prepared MoS_2_ nanosheets. The peaks at 232.6, 229.4 and 226.5 eV, corresponded to Mo^4+^3d_3/2_, Mo^4+^ 3d_5/2_ and S 2s_,_ respectively. In the S2p spectrum, S 2p_1/2_ and S 2p_3/2_ peaks also appeared at 163.5 eV and 162.3 eV, which is consistent with previously published papers^35–37^. These results show that the dominant 2H phase in the MoS_2_ nanosheets have been obtained from sonication-assisted exfoliation of MoS_2_ powder.

Moreover, the optical properties of MoS_2_ dispersion were investigated by UV-visible spectra. Fig. S2a shows the UV-visible spectrum of the diluted MoS_2_ dispersion in ethanol. As seen, the characteristic absorption bands appeared at approximately 400, 450, 610 and 670 nm. The two absorption peaks at about 610 and 670 nm were caused by A1 and B1 direct excitonic transition at the K point with energy separation. The peaks at 400 and 450 nm could be ascribed to the direct transition of M point from the deep valence band to the conduction band. All these results are in good agreement with the reported literatures^38,39^.

A fluorescence experiment was also conducted to verify that the prepared MoS_2_ was a structure of nanosheet. As is well known, the oligonucleotides of DNA can adsorb on the surface of the layered 2D TMDCs including MoS_2_, WS_2,_ etc, via van der Waals interactions, and subsequently the layered 2D TMDCs could quench the fluorescence of single-stranded DNA due to fluorescence resonance energy transfer (FRET). However, pristine TMDCs powder can’t quench the fluorescence of single-stranded DNA^40,41^. Fig. S2b shows strong fluorescent emission at the wavelength of approximately 520 nm for the FAM fluorophore-labeled DNA (FAM-DNA). With the addition of the as-prepared MoS_2_, up to 97% quenching of the fluorescent emission was observed, showing that MoS_2_ could effectively quench the fluorescence of FAM-DNA. The result further indicates that the obtained MoS_2_ nanosheet is layered nanomaterial of high quality. Figure S3a represents typical Raman spectra of RGO, in which the D band at 1350 cm^−1^ and the G band at 1600 cm^−1^ were displayed, respectively. The Raman spectra results of MoS_2_ nanosheets show two characteristic peaks at 383 and 407 cm^−1^, respectively. The strong Raman peaks of the MoS_2_ nanosheets suggest that the exfoliated MoS_2_ nanosheets are of high quality.

The SEM image was employed to characterize the prepared MoS_2_ FET device. As shown in Fig. 2e, it was seen that a few layer RGO sheet was laid flat in the channel and was connected to a pair of Au electrodes. After MoS_2_ nanosheets were drop-casted on the surface of RGO, the dense MoS_2_ nanosheets were well-proportionally distributed on the sensing channel (Fig. 2f). All the SEM results demonstrate the successful fabrication of MoS_2_/RGO FET device.

### Electrical properties of MoS_2_/RGO FET device

**Figure 3:**
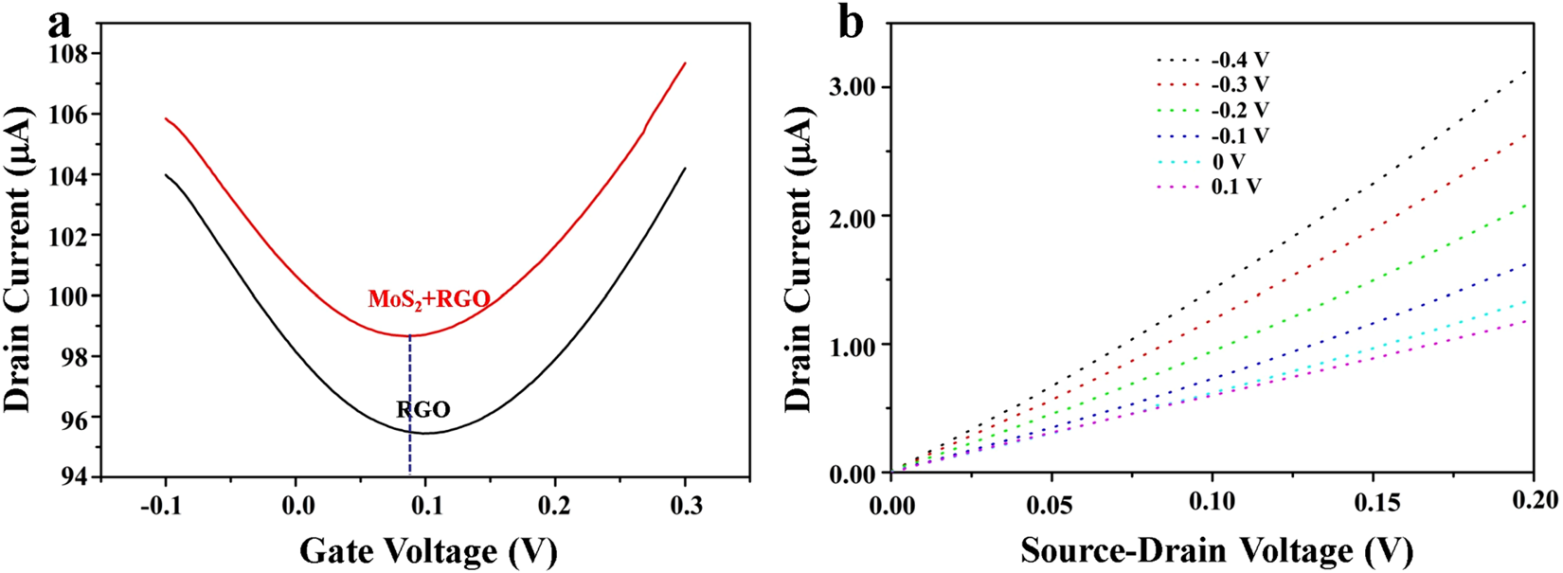
(**a**) Transfer characteristic curves of the RGO-based FET device (black line) and MoS_2_/RGO FET device (red line). (**b**) The output curves of the MoS_2_/RGO FET device at different V_GS_ value.

Current-voltage (I-V) curves were used to characterize the electrical properties of the MoS_2_/RGO-based FET device. As shown in Fig. 3, the transfer characteristic curves of the device were measured with RGO and MoS_2_/RGO FET devices, respectively. The ambipolar characteristics could be clearly observed at a small range of gate voltage (from −0.1 to 0.3 V) under ambient conditions. The dirac point of RGO transfer curve was found to be 0.1 V (Fig. 3a). After drop-casting MoS_2_ onto the surface of RGO, the Dirac point of the device shifted to left (0.08 V). When gate bias of Vg = 0.1 V was applied, the device showed n-type doping, indicating that the electronic conduction is dominant in graphene channels. This phenomenon is consistent with those reported by the literatures^42–44^. The I_ds_-V_ds_ curve was obtained to further examine the electrical characteristics of the MoS_2_/RGO FET device in Fig. 3b. The drain-source current decreased with a slight reduction of the gate voltage, indicating that the device response was sensitive to the gate voltage.

### Real-time electrical detection of H_2_O_2_

**Figure 4:**
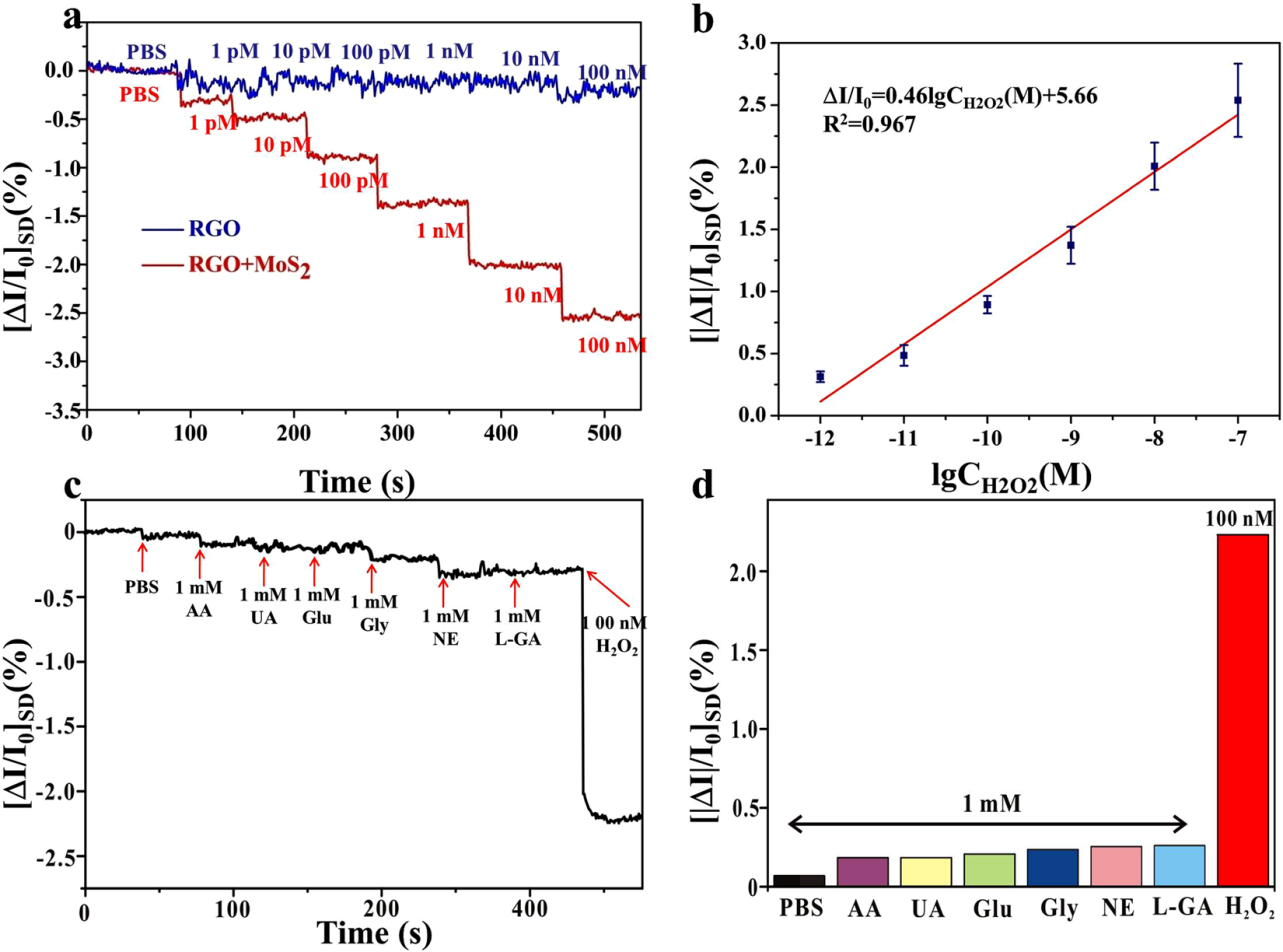
(**a**) Real-time detection of H_2_O_2_ with increasing concentrations in PBS buffer with the MoS_2_/RGO FET sensor (red line) and the RGO FET sensor (blue line). (**b**) The calibration curve of MoS_2_/RGO FET sensor to a series of H_2_O_2_ concentrations. Error bars represent standard deviations of measurements (n = 3). (**c**) Selectivity measurement with the addition of a series of interferents (PBS, 1 mM AA, 1 mM UA, 1 mM Glu, 1 mM GLY, 1 mM NE, 1 mM L-GA,) followed by 1 μM H_2_O_2_ solutions. (**d**) Histogram of the current change of the MoS_2_/RGO sensor to PBS, AA, UA, Glu, GLY, NE, L-GA and H_2_O_2_, respectively.

The sensitivity of the MoS_2_/RGO FET sensor was investigated by applying the freshly prepared H_2_O_2_ solutions of increasing concentrations ranging from 1 pM to 100 nM to the sensor, and the real time measurements were recorded. The changes of I_SD_ were used to monitor the responses of the MoS_2_/RGO FET sensor upon addition of H_2_O_2_ at various concentrations in real time. The sensor response to H_2_O_2_ was quantified using the normalized current changes (ΔI/I_0_ = (I_SD_–I_0_)/I_0_), where I_0_ is the initial current and I_SD_ is the stabilized current after changing the concentration of H_2_O_2_. As shown in Fig. 4a, the I_SD_ of FET sensor showed a gradual decrease as the concentration of H_2_O_2_ increased. The sensing mechanism may be attributed to generation of the positive charges upon addition of H_2_O_2_, leading to a conductance decrease of the MoS_2_/RGO FET sensor. As reported, MoS_2_ can play as peroxidase mimics^22,45^ for decomposing H_2_O_2_. For horseradish peroxidase (HRP), the reaction mechanism with H_2_O_2_ is to form the reactive enzyme intermediate compound and produce hydroxyl radicals and H^+^^46^. So the possible mechanism of MoS_2_ catalyzing H_2_O_2_ is similar to that of HRP. In such a case, a positive charge (H^+^) is generated when H_2_O_2_ is applied to the MoS_2_/RGO sensor. This is in good agreement with the literature, in which H_2_O_2_ can react with polypyrrole (PPy) to generate a positive charge on RGO/PPy nanotube FET-type sensor^47^. Furthermore, the applied gate bias of Vg = 0.1 V was less than the oxidation potential of H_2_O_2_ (>0.3 V), indicating that electrochemical oxidation of H_2_O_2_ did not take place on the sensor device, and the I_SD_ did not come from the oxidation of H_2_O_2_. This means that the reaction of catalytic decomposition of H_2_O_2_ occurred on the surface of MoS_2_/RGO. As discussed above, since MoS_2_ is a sandwich structure composed of two sulfur atoms and one molybdenum atom, protons can be penetrated to the middle layer, and the improvement in catalytic performance is probably due to the activity enhancement of the active sites in MoS_2_ by the intercalated protons^48^. Because of its intrinsic structural characteristics, MoS_2_ can act as peroxidase mimics for decomposing H_2_O_2_, wherein it produces positive charges in the process of catalysis. The positive charges were then bound to the surface of RGO, thereby attracting their counterions in graphene and inducing n-type doping. As the concentration of H_2_O_2_ rose, more positive charges were generated and bound to the surface of the graphene, and the carrier concentration decreased correspondingly, leading to the decreased current. The mechanistic scheme is displayed as Fig. S4. Figure 4b shows the current change ratio (ΔI/I_0_) versus different concentrations of H_2_O_2_. The H_2_O_2_ concentration showed a linear response to the drain current change ratio. The linear relationship was described as |ΔI| /I_0_ = 0.46lgC_H2O2_ + 5.66 (the logarithmic value of H_2_O_2_ concentration defined as lgC). The as-prepared MoS_2_/RGO FET sensor was extremely sensitive to H_2_O_2,_ and the limit of detection could be achieved down to 1 pM with the signal-to-noise ratio >3 (the noise level of the FET sensors was estimated by using PBS as baseline). The MoS_2_/RGO FET sensor shows the highest sensitivity compared with other H_2_O_2_ sensors, as shown in Table S1. The amazing sensitivity can be ascribed to both high conductivity of the RGO-based FET biosensor and high catalytic capability caused by MoS_2_ nanosheets. The positive charges generated in catalytic reaction could be sensitively detected by the MoS_2_/RGO FET, resulting in the conductance decrease of the FET sensor. Moreover, it was observed that the typical response time of this FET sensor to H_2_O_2_ was estimated to be less than 1 s, exhibiting that the FET sensor had a fast response. For comparison, the response of the RGO FET sensor (without MoS_2_) toward various concentrations of H_2_O_2_ (from 1 pM to 100 nM) was also investigated. The current change was negligible when different concentrations of H_2_O_2_ were added. An evident I_sd_ decrease was seen till the concentration of H_2_O_2_ reached 100 nM (blue line, Fig. 4a). This further implies that MoS_2_ is able to decompose H_2_O_2_ effectively, thereby producing a positive charge. Furthermore, more charge carrier density is formed on the MoS_2_/RGO FET device than the RGO FET device, making the larger readable signal in current change at low H_2_O_2_ concentration range.

The specificity of the MoS_2_/RGO FET sensor towards H_2_O_2_ was further investigated by real-time recording I_SD_ upon addition of a series of interfering species in 1 × PBS solution, including ascorbic acid (AA), uric acid (UA), glutamate (Glu), glycine (GLY), Noradrenaline hydrochloride (NE), L-glutamine (L-GA). As show in Fig. 4c, when 1 × PBS, 1 mM AA, 1 mM UA, 1 mM Glu, 1 mM GLY, 1 mM NE, 1 mM L-GA, respectively, were successively introduced to the MoS_2_/RGO FET sensor, negligible current change was observed. However, when 100 nM H_2_O_2_ was injected, a remarkable current response was observed, even in the case that interfering species of high concentration coexisted in the analyte. To directly demonstrate the response difference, current changes of the various substances were summarized (Fig. 4d). These results firmly exhibit high specificity of the MoS_2_/RGO FET sensor toward H_2_O_2_. Then, the repeatability and stability tests were also conducted to illustrate the excellent property of the MoS_2_/RGO FET sensors by using more than 3 sensors, respectively. Firstly, for stability test, the as-prepared MoS_2_/RGO FET device was stored in a vacuum oven for 3, 7, 10 and 14 days, respectively, then used for the detection of 100 nM H_2_O_2_. As described in the Fig. S5a, the shift of the dirac voltage was only changed 12.5% compared with its original value over 2 weeks. This signal decrease may be caused by the nonspecific surface adsorptions. Secondly, the repeatability of the MoS_2_/RGO FET sensors was also evaluated. One MoS_2_/RGO FET sensor was chosen to repeatedly detect 100 nM H_2_O_2_ concentration for 7 times, as shown in the Figure S5b. The dirac voltage change of the sensor remained nearly the same after 7 measurements and a relative standard deviation (RSD) was 2.1%. The results demonstrate high repeatability of the sensor.

### Real-time monitoring of H_2_O_2_ released from HeLa cells

**Figure 5:**
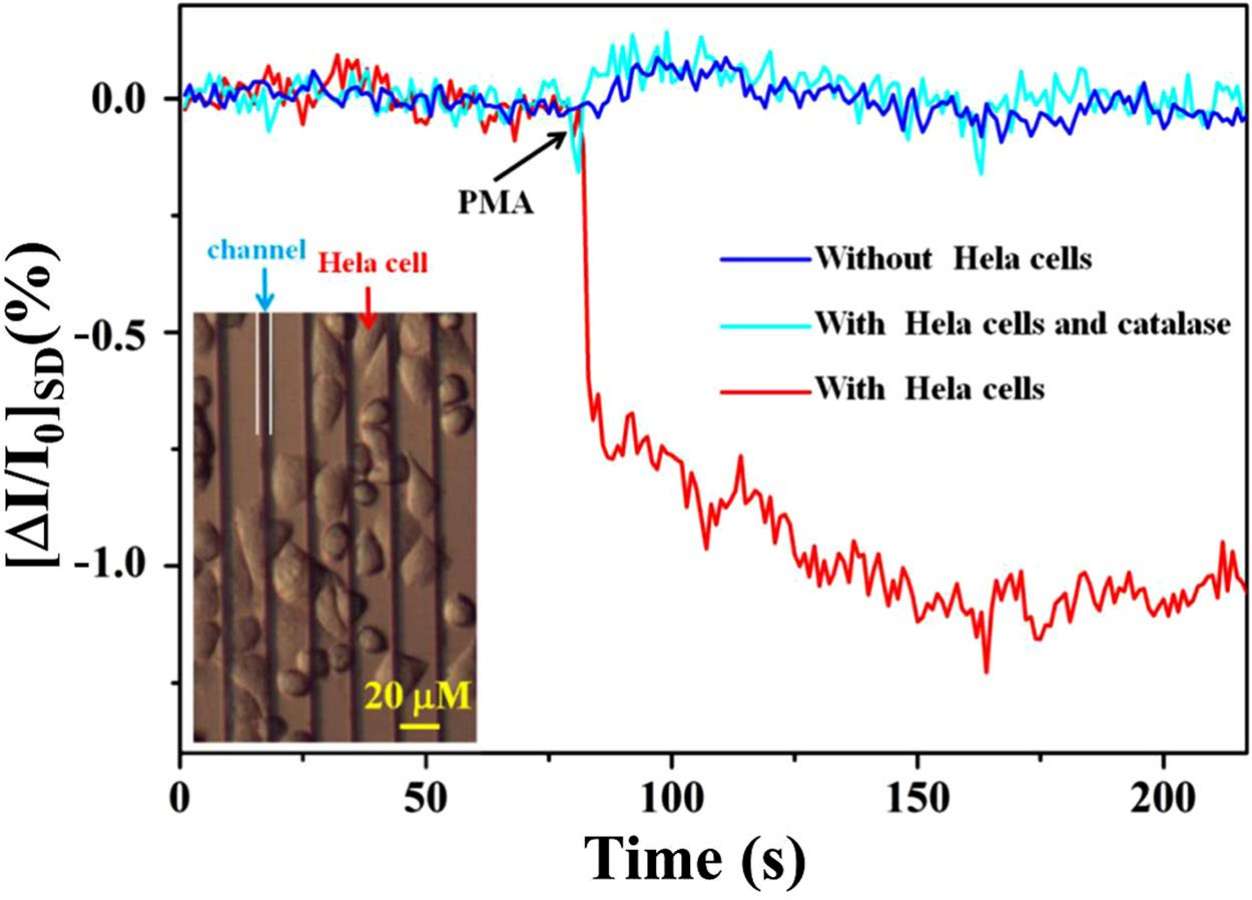
Real-time current response of the MoS_2_/RGO FET sensors toward H_2_O_2_ after PMA was added into PBS buffer solution in the presence of HeLa cells (red line) and in the absence of HeLa cells (blue line).The purple line shows the case that H_2_O_2_ scavenger catalase was mixed with the PBS solution and added onto the devices containing HeLa cells. Inset: Optical microscope image shows the HeLa cells grown well on the MoS_2_/RGO FET sensor array.

H_2_O_2_ plays a significant role in many cell functions and can be used as potential marker for tumor cells. Consequently, it is very meaningful to sensitively detect H_2_O_2_ from living cell because of its diverse biological functions. For this experiment, we first investigated the influence of weak acid environment on sensor’s performance from pH 6.4 to 7.4 (These different pH values were made by adding HCl or NaOH to PBS solutions, which were finally adjusted by a commercial pH meter). The results show that the weak acid environment did not have significant influence on the change of Dirac point after different pH values were applied (Figure S6). Then, real-time detection of extracellular H_2_O_2_ released from HeLa cells was performed by the MoS_2_/RGO FET sensor. As known, the HeLa cells can generate H_2_O_2_ when stimulated by phorbol 12-myristate 13-acetate (PMA, PMA is a potent activator of protein kinase C (PKC), which can activate PKC to produce H_2_O_2_). On the contrary, H_2_O_2_ can be decomposed by catalase. Before cell level measurement, HeLa cells were cultured for 24 h on the MoS_2_/RGO FET sensor surface by using a self-made plastic culture chamber. After cell culture, the cells were found to be in good condition, as seen in Fig. 5, Inset. Afterwards, the culture medium was replaced with the same amount of PBS solution. As shown in Fig. 5, when PMA (with the final concentration of 1 μg/mL) was added into the cell chamber, the current declined immediately and then slowly stabilized in a short time (red line). Based on the current change generated in Fig. 5, we could semi-quantify the released H_2_O_2_ from cells by the working curve in Fig. 3b. The H_2_O_2_ concentration was then calculated to be about 100 pM. On the contrary, when the catalase (300 U/mL, H_2_O_2_ scavenger) was mixed with the test solution, hardly any current change was obtained upon addition of PMA to HeLa cells (purple line). The catalase can metabolize H_2_O_2_ to water and oxygen, so hardly H_2_O_2_ could be monitored by the MoS_2_/RGO FET sensor. For control experiments, PMA was added to the MoS_2_/RGO surface without culturing the HeLa cells. On this occasion, barely any current change was obtained (blue line). The above-mentioned experiment results prove that the observed current response indeed came from H_2_O_2_ released from the HeLa cells. These results firmly demonstrate that the constructed MoS_2_/RGO FET sensor was capable of real-time monitoring H_2_O_2_ released from living cells.

## Conclusions

In conclusion, we have constructed the MoS_2_/RGO FET sensor capable of monitoring H_2_O_2_ release from cancer cells in a highly sensitive manner. The MoS_2_ nanosheets were prepared by a simple liquid ultrasonication exfoliation method and the MoS_2_/RGO FET sensors were fabricated by drop-casting the MoS_2_ nanosheets to the RGO FET device. Compared to the previously published works regarding various sensors for H_2_O_2_ detection, our FET-type sensor showed a rapid response to the changes of H_2_O_2_ concentration and an ultrahigh sensitivity to 1 pM. In addition, the FET sensor also demonstrated high specificity toward H_2_O_2_ in the presence of AA, UA, Glu, GLY, NE, L-GA. Moreover, the device provided an enzyme-free detection platform for the real-time detection of H_2_O_2_ released from HeLa cells. This work opens a new way for constructing nonenzymatic FET sensors for detecting ROS released from cells, and helps understand the mechanism of H_2_O_2_ sensing by MoS_2_. From the perspective of sensor performance, the MoS_2_/RGO FET can be used as alternative methods for the detection of H_2_O_2_ in many fields, and may play a significant role in the clinical detection of H_2_O_2_-related diseases.

## Methods

### Materials

N-methyl-2-pyrrolidone (NMP), uric acid (UA), phorbol 12-myristate 13-acetate (PMA), glycine (GLY), ascorbic acid (AA), glutamate (Glu), Noradrenaline hydrochloride (NE), L-glutamine (L-GA) and catalase were purchased from Sigma-Aldrich (St. Louis, MO, USA). The pristine MoS_2_ powder and graphite flake powder used in the experiments were purchased from Nanjing XFNANO Materials Tech. Co. Ltd. (Nanjing, China). Ultrapure water was generated from Millipore water purification system (18.2 MΩ.cm resistivity, Milli-Q Direct 8). Hydrogen peroxide (H_2_O_2_, 30%), and other chemical reagents were purchased from Sinopharm Chemical Reagent Co. Ltd. (Beijing, China). PBS buffer solution used in this work was pH = 7.4.

### Preparation of MoS_2_ nanosheets

The MoS_2_ nanosheets were prepared using a simple liquid ultrasonication method^49–52^. Briefly, 1 g of pristine MoS_2_ powder was added to 100 mL flask, after which 50 mL NMP was added to the flask as dispersion solvent. The mixture was sonicated (Power: 200 W, Frequency: 20 KHZ) until to obtain a black homogeneous suspension at room temperature (usually needs 10 h). After that, the resultant dispersions were centrifuged for 30 min at 2000 rpm and then the top 2/3^th^ part of the supernatant were decanted. To remove NMP and determine the concentration of the MoS_2_ nanosheets in the dispersion solvent, the dispersion was vacuum filtered through a nylon membrane with a pore size of 0.22 μm, followed by washing the membrane with large amount of distilled water and ethanol. The resultant film was dried for 24 h at 60 °C in vacuum oven. The MoS_2_ nanosheets powder was then peeled from the resultant film.

### Fabrication of the MoS_2_/RGO sensor

The RGO-based FET device was produced by the previously reported method^25^. Firstly, 10 mg of GO was added to 10 mL of 98% hydrazine followed by sonication for 45 min to produce a black suspension of hydrazinium graphene, and the suspension was placed for 1 week to obtain the thorough reduction of GO. The resulting RGO suspensions could be stable for months with little aggregation. To construct the RGO layer on the pre-fabricated FET chip, diluted RGO suspension (0.15 mg/ml) was drop-casted onto the channel and thermally annealed at 80 °C for 2 h in order to enhance the contact between the RGO and the electrodes. In order to prepare the MoS_2_/RGO FET sensor, the MoS_2_ nanosheets powder was dispersed into ethanol to form MoS_2_ nanosheets dispersion with the concentration of 1 mg/mL. Then MoS_2_ dispersion was drop-casted on the surface of RGO. The whole device was thermally annealed at 80 °C for 0.5 h in vacuum oven for enhancing the contact among the MoS_2_, RGO and electrodes.

### Electrical detection of H_2_O_2_ in PBS solution

Electrical detection of H_2_O_2_ was monitored in a liquid gate environment in real time under a constant bias voltage of 10 mV and liquid gate of 0.1 V. As described in our previously published papers^25–28^, a silver wire was used as the liquid gate in this work. When the measurement was performed, the silver wire was immersed in buffer solution. The different concentrations of H_2_O_2_ were determined by diluting H_2_O_2_ (30%) using 1 × PBS buffer solution. H_2_O_2_ was manually added to the detection chamber with a gradually increasing concentration ranging from 1 pM to 100 nM for the sensitivity experiment. The specificity experiment was conducted using the same method for discriminating different interferents.

### Cell Culture

HeLa cells were purchased from cell bank of Xiangya Medical College (Changsha, China). They were routinely cultured in Dulbecco’s Modified Eagle Medium cell culture medium containing 10% fetal bovine serum (FBS) in a culture flask and supplemented with 1% penicillin at 37 °C, 5% CO_2_. HeLa cells were digested by trypsin from culture flask after growing to 90% confluence.

### Electrical detection of H_2_O_2_ released from HeLa cells

For real-time detection of H_2_O_2_, a self-made liquid reservoir was mountained on the sensing channel (Fig. S7). After that, the total device was sterilized for 30 min via ultraviolet in a biosafety cabinet. Then the sensor was used for cell culture experiments. HeLa cells were seeded on the MoS_2_/RGO sensors confined in a self-made liquid reservoir at a density of ~1 × 10^4^ cell/cm^2^. After 10 h of incubation, cells were used for stimulation and detection. Upon detection, the culture medium was then changed by the 1 × PBS. After reaching a steady-state baseline, PMA (1 μg/mL) as the H_2_O_2_ stimulant was introduced into the self-made liquid reservoir and H_2_O_2_ released from HeLa cells was detected by the real-time working mode present in the form of changes in current. Then catalase (300 U/mL) was injected into the liquid reservoir for the purpose of degrading H_2_O_2_, as the control experiment. The electrical measurement condition was the same as above described.

### Instrumentation

The morphology of the as-prepared MoS_2_ was characterized by TEM (JEOL JEM-2100, Japan) operated at 200 kV. The MoS_2_ dispersion was further diluted with ethanol and dropped on a carbon-coated film copper grid for subsequent TEM observation. The X-ray photoelectron spectroscopy (XPS) analysis was conducted using an ESCALAB 250 Xi XPS system (Thermo Fisher Scientific, American). UV-visible spectra were measured by UV-2550 (Shimadzu Co. Ltd., Japan). Raman spectra were taken by using Invia Renishaw spectrometer (RM 1000, England) equipped with 514.5 nm laser line. X-ray diffraction (XRD) analysis was conducted on PANalytical X’Pert Pro diffractometer (PANalytical, Holland). The fluorescence spectra were obtained by a Hitachi F-4600 spectrophotometer (Hitachi Co. Ltd., Japan). Scanning electron microscopy (SEM) images were obtained on a field-emission scanning electron microscope (Zeiss, Germany). All electrical measurements were recorded with a Keithley 4200 semiconductor characterization system and a shield probe station (Everbeing BD-6, Taiwan).

## Electronic supplementary material

Sensitive Molybdenum Disulfide Based Field Effect Transistor Sensor for Real-time Monitoring of Hydrogen Peroxide
