# Supplementary material for: Sensitive Molybdenum Disulfide Based Field Effect Transistor Sensor for Real-time Monitoring of Hydrogen Peroxide
Source: Sci Rep. 2019 Jan 24;9:759. doi: 10.1038/s41598-018-36752-y (PMC6345991; doi:10.1038/s41598-018-36752-y)
Supplement: Supplementary file 1 — Sensitive Molybdenum Disulfide Based Field Effect Transistor Sensor for Real-time Monitoring of Hydrogen Peroxide [file 41598_2018_36752_MOESM1_ESM.doc]

**Supporting Information**

**Sensitive Molybdenum Disulfide Based Field Effect Transistor Sensor for Real-time Monitoring of Hydrogen Peroxide**

Chao Zheng^1, 2, [+]^, Xin Jin^1, [+]^, Yutao Li^1,*^, Junchi Mei^1^, Yujie Sun^1^, Mengmeng Xiao^3^, Hong Zhang^4^, Zhiyong Zhang^3,*^, Guo-Jun Zhang^1,*^

^1^School of Laboratory Medicine, Hubei University of Chinese Medicine, 1 Huangjia Lake West Road, Wuhan 430065, P.R.China

^2^Department of Medical Laboratory, The Central Hospital of Wuhan,Tongji Medical College, Huazhong University of Science and Technology, Shengli Street Jiang’an District No.26,Wuhan 430014,P.R.China

^3^Key Laboratory for the Physics and Chemistry of Nanodevices, Department of Electronics, Peking University, No.5 Yiheyuan Road Haidian District, Beijing 100871, P.R.China

^4^Teaching and Research Office of Forensic Medicine, Hubei University of Chinese Medicine, 1 Huangjia Lake West Road, Wuhan 430065, P.R.China

^[+]^These authors contributed equally to this work

^*^Corresponding author: Tel: +86-27-68890259, Fax: +86-27-68890259

E-mail: [zhanggj@hbtcm.edu.cn](mailto:zhanggj@hbtcm.edu.cn); [zyzhang@pku.edu.cn](mailto:zyzhang@pku.edu.cn); [liyutaokuaile29@163.com](mailto:liyutaokuaile29@163.com)


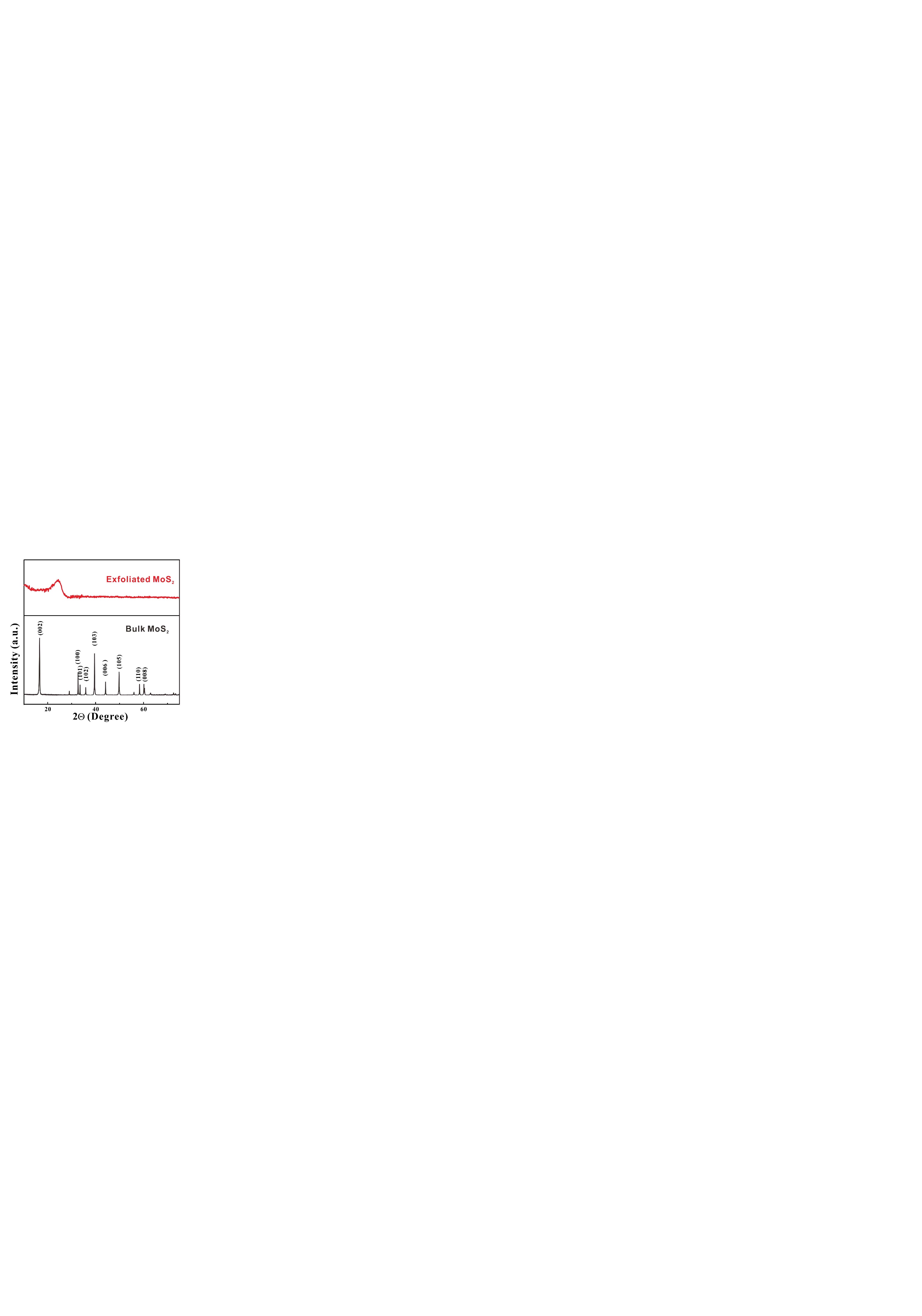


Fig. S1. XRD patterns of the exfoliated MoS_2_ nanosheets and the bulk MoS_2_ crystallite.


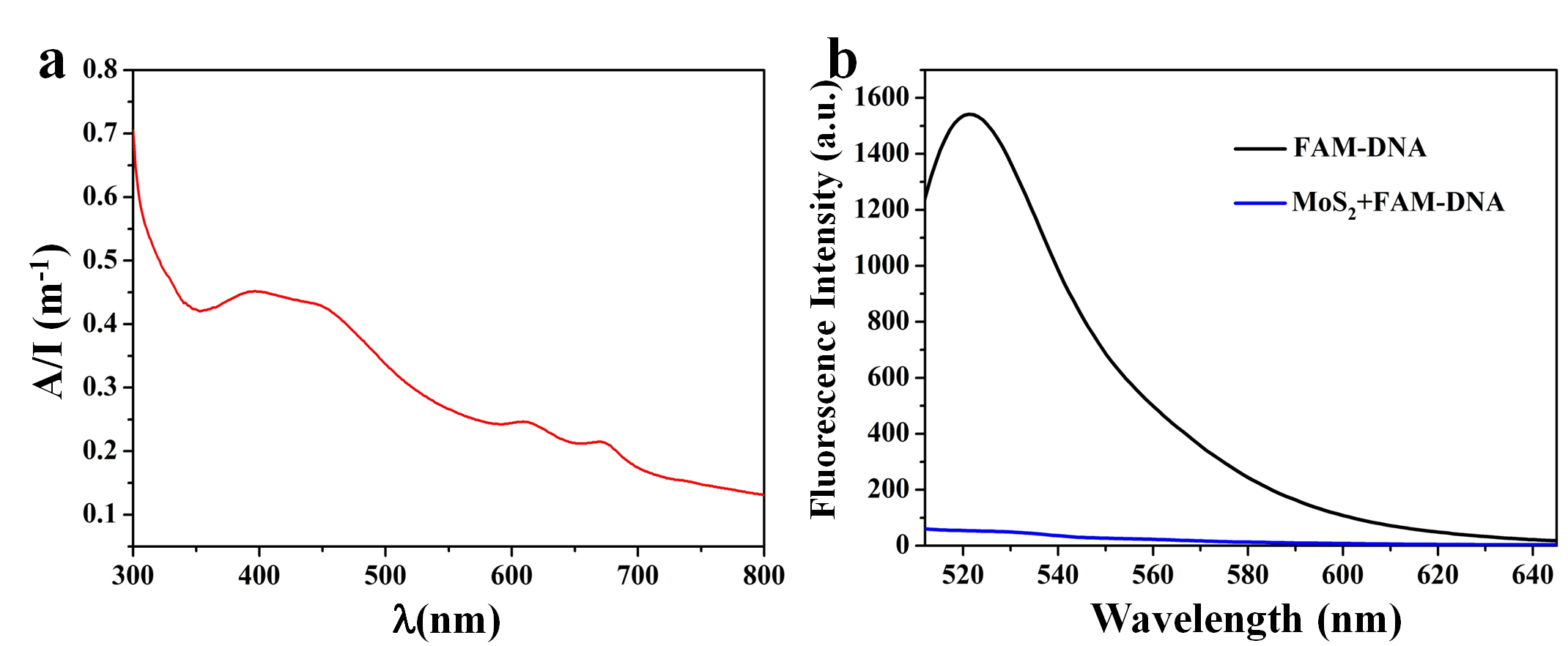


Fig. S2. (a) UV-visible adsorption spectrum of the MoS_2_ nanosheets dispersed in ethanol. (b) Fluorescence spectra of FAM-DNA in the absence and presence of MoS_2_ nanosheets.


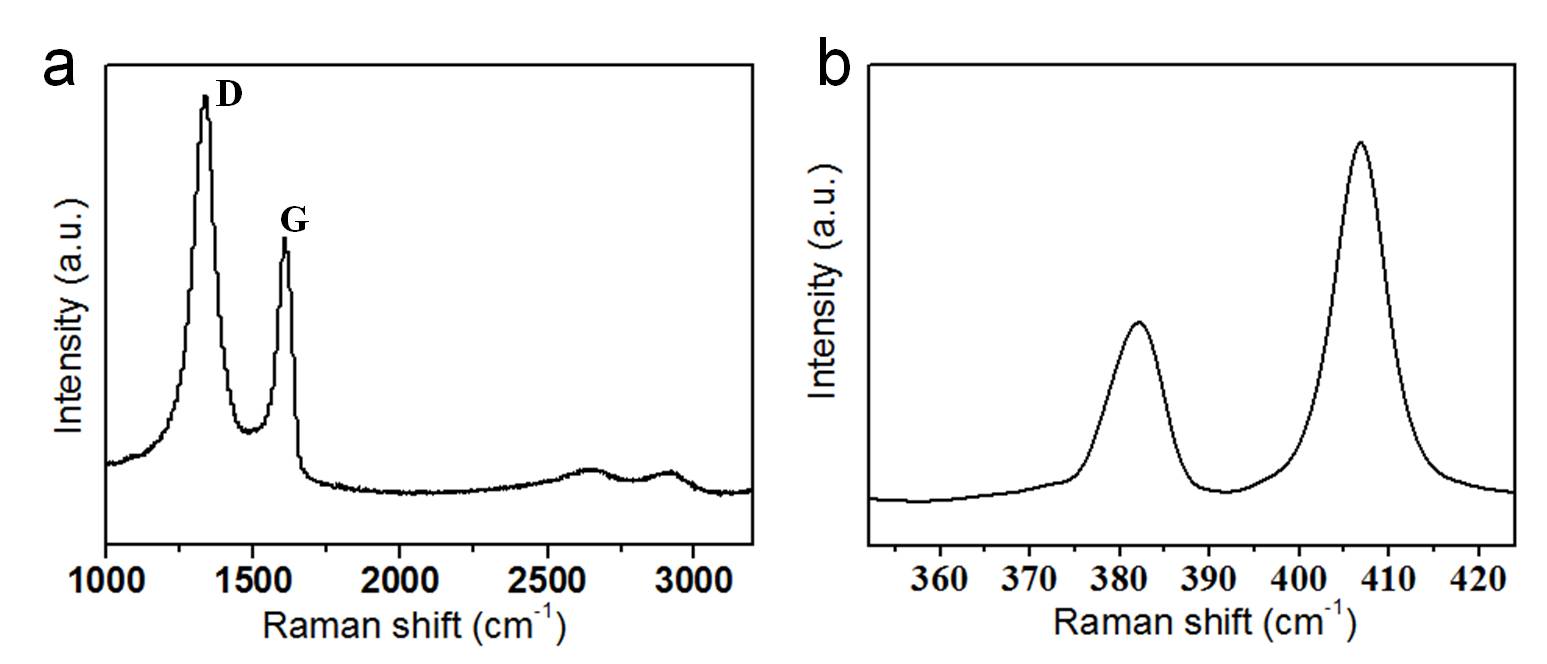


Figure S3. Raman spectra of RGO (a) and MoS_2_ nanosheets (b)


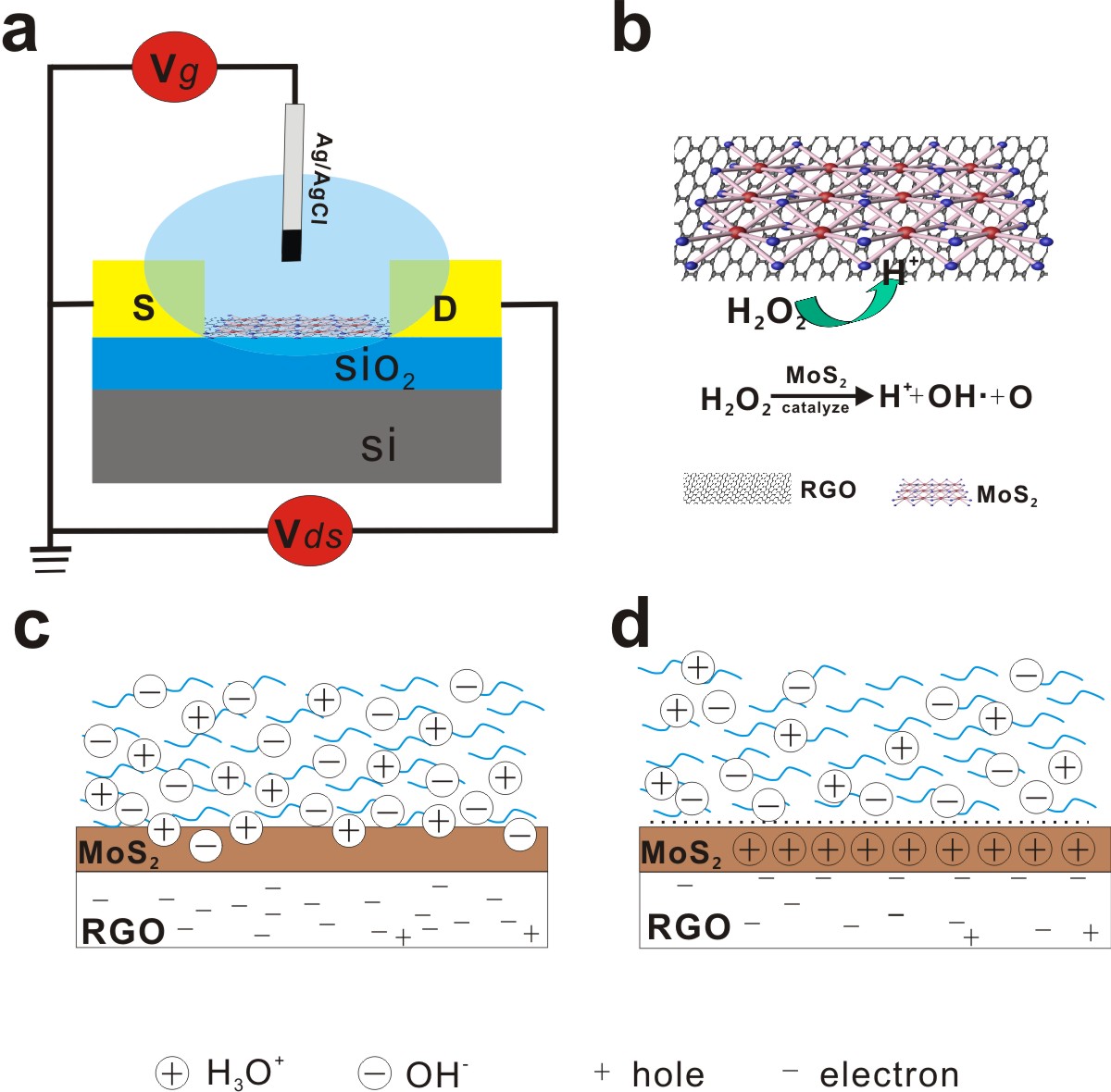


Fig. S4. (a) Schematic diagram of H_2_O_2_ FET sensor, in which a silver wire was used as the liquid gate. (b) A possible mechanism of the MoS_2_/RGO nanocomposites for catalytically decomposing H_2_O_2_. (c) Distribution of ions and donors (holes or electrons) at the MoS_2_/RGO-electrolyte solution. (d) Distribution of ions and donors (holes or electrons) at the graphene-electrolyte solution in the presence of positive charge on the MoS_2_/RGO surface.


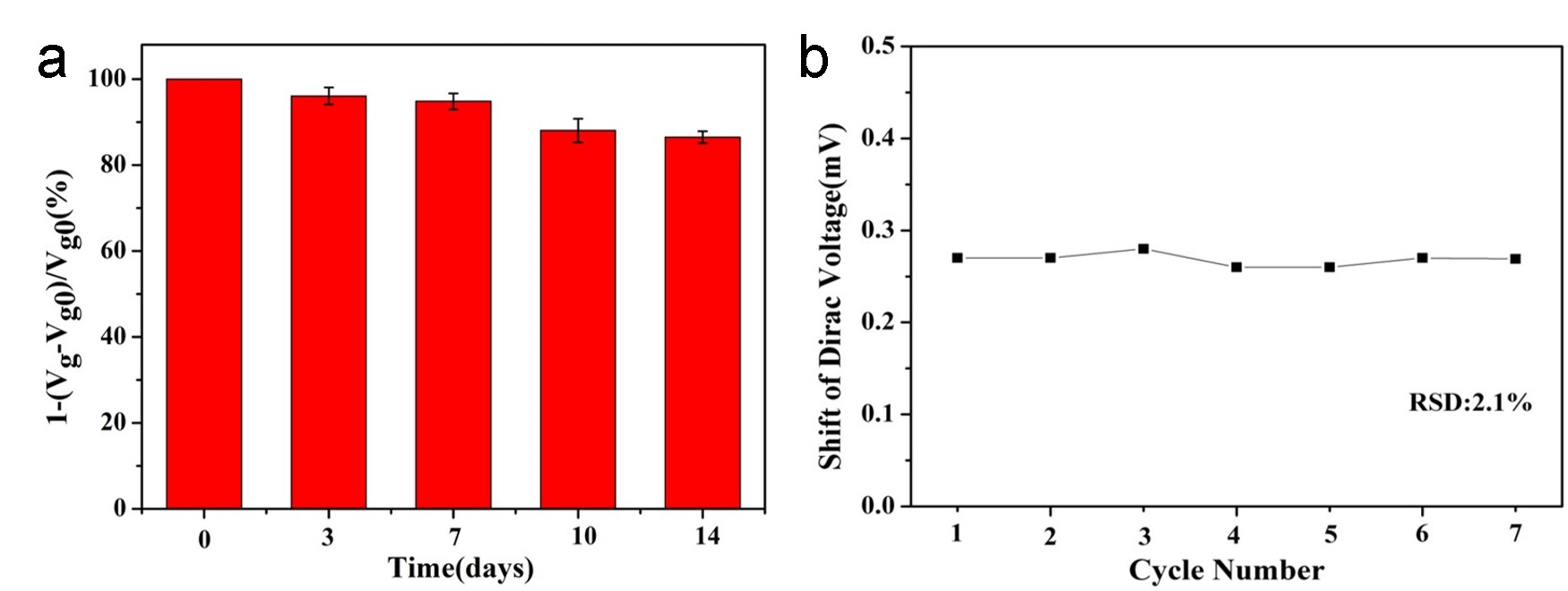


Fig. S5. (a) Stability test for the MoS_2_/RGO FET device over 2 weeks. (b) Repeatability of the MoS_2_/RGO FET device for detecting 100 nM H_2_O_2_ concentration for 7 times.


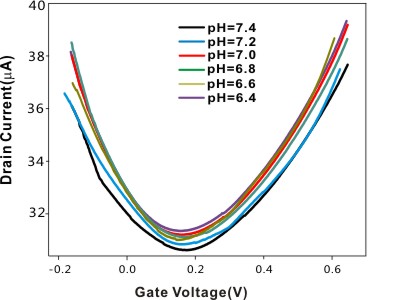


Figure S6. Influence of weak acid environment on sensor’s performance from pH 6.4 to 7.4.


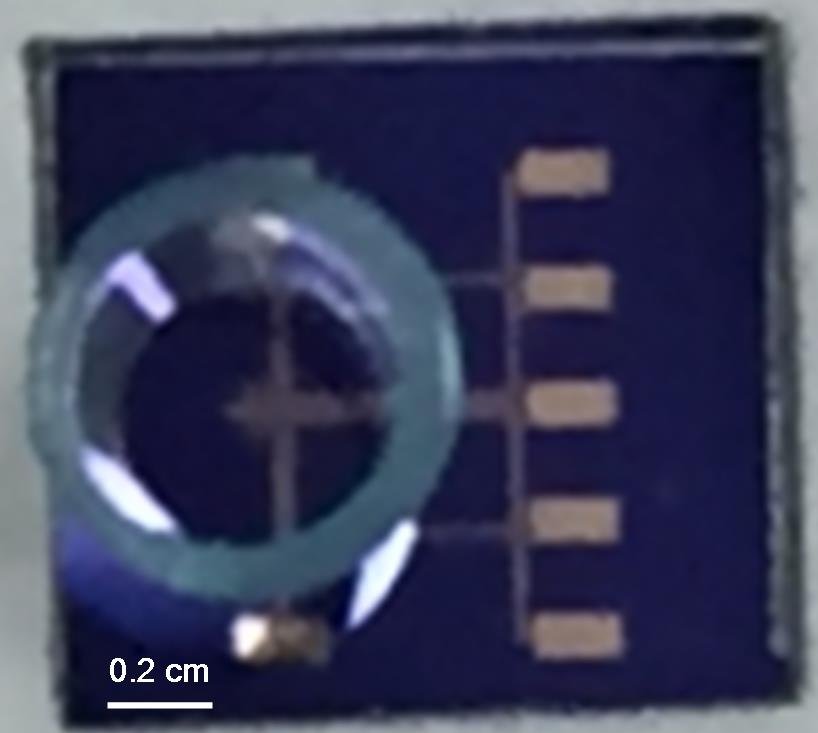


Fig. S7. Optical image of the FET device with a self-made liquid reservoir.

Table S1. Comparison of various H_2_O_2_ sensors

| Electrode Materials | Methods | Detection limit | Ref |
| --- | --- | --- | --- |
| AgNP/CNT | Electrochemistry | 0.5 μM | 1 |
| AgNP/ZnO | Electrochemistry | 0.42 μM | 2 |
| MnO_2_/GO | Electrochemistry | 0.8 μM | 3 |
| Pt/graphene | Electrochemistry | 0.2 μM | 4 |
| Fe_3_O_4_/rGO | Electrochemistry | 6 nM | 5 |
| AuCu NWs | Electrochemistry | 2 nM | 6 |
| MoS_2_ NP | Electrochemistry | 2.5 nM | 7 |
| PPy NT/rGO | FET | 100 pM | 8 |
| MoS_2_/rGO | FET | 1 pM | This work |

NP: nanoparticle, CNT: carbon nanotube, GO: graphene oxide, rGO: reduced grapheme oxide, NWs: nanowires, NT: nanotube.

**REFERENCE:**

1. Wei, Z. *et al*. A novel nonenzymatic hydrogen peroxide sensor based on multi-wall carbon nanotube/silver nanoparticle nanohybrids modified gold electrode. *Talanta* **80**, 1029-1033 (2009).

2. Wang, Q. & Zheng, J. Electrodeposition of silver nanoparticles on a zinc oxide film: improvement of amperometric sensing sensitivity and stability for hydrogen peroxide determination. *Microchim. Acta* **169**, 361-365 (2010).

1. Li, L. *et al*. A novel nonenzymatic hydrogen peroxide sensor based on MnO_2_/graphene oxide nanocomposite. *Talanta* **82,** 1637-1641 (2010).
2. Zhang, Y. *et al*. Highly sensitive graphene-Pt nanocomposites amperometric biosensor and its application in living cell H_2_O_2_ detection. *Anal. Chem.* **86**, 9459-9465 (2014).

5. Teymourian, H., Salimi, A. & Khezrian, S. Fe3O4 magnetic nanoparticles/reduced graphene oxide nanosheets as a novel electrochemical and bioeletrochemical sensing platform. *Biosens. Bioelectron.* 49C, 1-8 (2013).

1. Wang, N., Han, Y., Xu, Y., Gao, C. & Cao, X. Detection of H_2_O_2_ at the Nanomolar Level by Electrode Modified with Ultrathin AuCu Nanowires. *Anal. Chem.* **87**, 457-463 (2015).
2. Wang, T. *et al*. Biosensor based on ultrasmall MoS_2_ nanoparticles for electrochemical detection of H_2_O_2_ released by cells at the nanomolar level. *Anal. Chem*. **85**, 10289-10295 (2013).
3. Park, J. W., Park, S. J., Kwon, O. S., Lee, C. & Jang, J. Polypyrrole nanotube embedded reduced graphene oxide transducer for field-effect transistor-type H_2_O_2_ biosensor. *Anal. Chem.* **86**, 1822-1828 (2014).
